# Translational aspects of deep brain stimulation for chronic pain

**DOI:** 10.3389/fpain.2022.1084701

**Published:** 2023-01-11

**Authors:** Rosana L. Pagano, Camila S. Dale, Ana Carolina P. Campos, Clement Hamani

**Affiliations:** ^1^ Laboratory of Neuroscience, Hospital Sírio-Libanês, São Paulo, Brazil; ^2^ Laboratory of Neuromodulation and Experimental Pain, Department of Anatomy, University of São Paulo, São Paulo, Brazil; ^3^ Sunnybrook Research Institute, Hurvitz Brain Sciences Centre, Toronto, ON, Canada; ^4^ Harquail Centre for Neuromodulation, Sunnybrook Health Sciences Centre, Toronto, ON, Canada; ^5^ Division of Neurosurgery, Sunnybrook Health Sciences Centre, University of Toronto, Toronto, ON, Canada

**Keywords:** deep brain stimulation, pain, animal models, clinical trials, thalamus, periaqueductal grey matter

## Abstract

The use of deep brain stimulation (DBS) for the treatment of chronic pain was one of the first applications of this technique in functional neurosurgery. Established brain targets in the clinic include the periaqueductal (PAG)/periventricular gray matter (PVG) and sensory thalamic nuclei. More recently, the anterior cingulum (ACC) and the ventral striatum/anterior limb of the internal capsule (VS/ALIC) have been investigated for the treatment of emotional components of pain. In the clinic, most studies showed a response in 20%–70% of patients. In various applications of DBS, animal models either provided the rationale for the development of clinical trials or were utilized as a tool to study potential mechanisms of stimulation responses. Despite the complex nature of pain and the fact that animal models cannot reliably reflect the subjective nature of this condition, multiple preparations have emerged over the years. Overall, DBS was shown to produce an antinociceptive effect in rodents when delivered to targets known to induce analgesic effects in humans, suggesting a good predictive validity. Compared to the relatively high number of clinical trials in the field, however, the number of animal studies has been somewhat limited. Additional investigation using modern neuroscience techniques could unravel the mechanisms and neurocircuitry involved in the analgesic effects of DBS and help to optimize this therapy.

## Introduction

Chronic pain is a major health problem associated with individual suffering and an important social and economic burden. The prevalence of this condition in the general population ranges from 8% to 50% (1–3). It is estimated that approximately 30% of chronic pain patients have neuropathic pain (4, 5). This may be defined as pain due to lesions and/or dysfunction of the nervous system. Though pharmacotherapy, physiotherapy and nerve blocks are often effective, the treatment of neuropathic pain may be quite challenging. This is in contrast to nociceptive pain, which is associated with physical damage to the body.

Deep brain stimulation (DBS) involves the delivery of electrical current to the brain parenchyma through implanted electrodes (6, 7). Its use for the treatment of pain was one of the first applications of this technique in Functional Neurosurgery (8–10). Despite being proposed approximately 70 years ago, it was only in the 1970s and 1980s that DBS became more widely studied for the treatment of pain, with stimulation being more frequently delivered to the periaqueductal (PAG)/periventricular gray matter (PVG), sensory thalamic nuclei, or the internal capsule (IC) (11, 12). More recently, with a better appreciation of the emotional components of pain, stimulation of the anterior cingulate cortex (ACC) (13–16) and the ventral striatum/anterior limb of the internal capsule (VS/ALIC) (17) has also been proposed.

In various applications of DBS, animal models have either provided the rational for clinical use or were utilized to study potential mechanisms of therapeutic responses (7, 18). Despite being conducted for over 50 years, the number of reports studying the effects of DBS in preclinical models may be considered limited compared to that of human publications.

In this review, we summarize the effects of DBS in preclinical models and in patients with chronic pain.

## Preclinical models

Prior to discussing preclinical work, it is worth differentiating nociception from pain. The former refers to the neural encoding of impending or actual tissue damage, whereas the later denotes the subjective experience of actual or impending harm. In general, preclinical models are largely suited to measure nociception. That said, animals are likely to have a subjective experience associated with nociception. Unfortunately, measuring this component is not straightforward. To decrease the gap with the human condition, proposed approaches include measures of spontaneous and evoked pain, pain memory, avoidance, anxiety- or depression-like behaviors, and the social modulation of pain (empathy).

**Figure 1:**
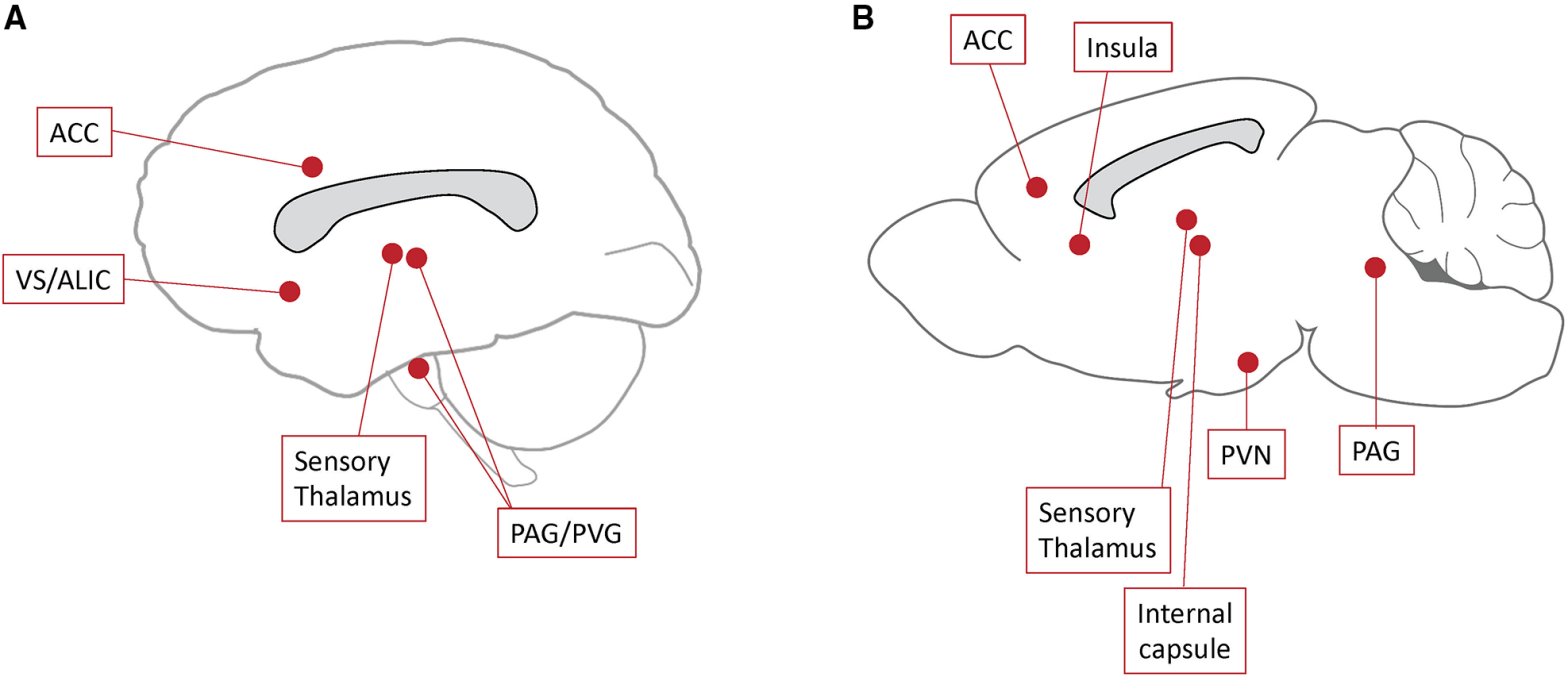
Deep brain stimulation (DBS) targets. Schematic representation of DBS targets studied in recent clinical trials (**A**) and preclinical models (**B**). ACC, anterior cingulum; PAG, periaqueductal gray matter; PVG, periventricular gray matter; PVN, paraventricular nucleus; VS/ALIC, ventral striatum/anterior limb of the internal capsule.

Structures targeted in preclinical DBS studies are either part of the pain matrix, limbic system, descending analgesic systems, or those potentially capable of modulating nociceptive signals (Figure 1). Itemized results according to target are presented below.

### Periaqueductal grey matter

The PAG is a highly conserved midbrain region that surrounds the cerebral aqueduct (19). It is a critical site for encoding aversive prediction errors and the processing of nociceptive information (19). The PAG receives strong nociceptive input from the spinal cord and provides top-down brainstem–spinal cord modulation to flexibly shape the experience of pain in different contexts, including the transition from acute to chronic states (19). Two separate nociceptive modulatory systems operate in the caudal PAG: a dorsal system, which encompasses its dorsomedial, dorsolateral and lateral subdivisions, and a ventral system that includes the ventrolateral PAG and dorsal raphe (20).

In an initial report, Reynolds (21) showed that PAG electrical stimulation produced analgesia in rats, opening a new research venue for the treatment of pain. Following that work, PAG electrical stimulation was considered a pain suppression method against various nocifensive behaviors (22, 23). In rats, electrical stimulation of the PAG inhibited spinal nociceptive transmission from the brainstem by activating descending inhibition mediated by opioids and glutamate (24). Analgesia-producing PAG stimulation alters the spontaneous activity of neurons in the medullary reticular formation (MRF) and inhibits the noxious-evoked excitation of MRF neurons. The microinjection of morphine into the PAG mitigated the reduced spontaneous activity of MRF units and inhibited noxious-evoked neuronal excitation. These effects were particularly observed in analgesia produced by PAG manipulations and were partially reversed by naloxone (25). PAG stimulation attenuated formalin-induced pain and increased the nociceptive threshold of complete Freund's adjuvant (CFA)-induced inflammatory pain (20, 26, 27). In addition, PAG stimulation has also been shown to inhibit visceral nociceptive responses of spinal dorsal horn neurons (28). Overall, the results described above suggest that electrical stimulation of the PAG works more effectively on nociceptive pain, including that related to acute and chronic inflammation.

### Sensory thalamus

Initial evidence for targeting the sensory thalamus in pain derived from the ablative neurosurgical literature (29–31). Sensory thalamic nuclei receive somatotopically oriented input from lemniscal fibers involved in sensory processing. Afferents from the face and body innervate the ventral posteromedial (VPM) and ventral posterolateral (VPL) nuclei, respectively. In rats with neuropathic pain, VPL-DBS reduced mechanical allodynia (27, 32–34), cold allodynia (35), and thermal hyperalgesia (36). Similar to humans, VPL-induced analgesia in rats with peripheral neuropathy was only documented when electrodes were implanted in a somatotopic manner (33). In inflammatory pain models, VPL stimulation was found to produce analgesia in chronic, but not acute preparations (27). Sensory thalamic stimulation also failed to reduce experimental pain in healthy animals (37, 38). These observations are well aligned with clinical findings suggesting that VPL-induced analgesia is more effective for the treatment of neuropathic pain (39–41).

The mechanisms through which thalamic DBS induces analgesia are still unclear. VPM-DBS inhibited the abnormal hyperactivity of nociceptive neurons in the trigeminal medullary dorsal horn of deafferented cats (42). In primates, sensory thalamic stimulation decreased the activation of spinothalamic tract neurons (43), an effect that seems to be partly mediated by corticofugal and descending inhibitory pathways (44). Corroborating these findings, VPL-DBS has been suggested to induce serotonin release in the spinal cord of primates *via* the modulation of raphe-spinal tract fibers (45). VPL stimulation has also been shown to inhibit local neurons and induce neuronal reorganization, modifying spontaneous pathological activity, and inhibiting nociceptive impulse transmission to cortical areas (46, 47).

### Internal capsule

In cats (48) and rats (49), electrical stimulation of IC fibers reduced thalamic nociceptive activity, having no effect on activity of dorsal horn neurons in response to noxious heat (50). IC stimulation inhibited neuronal hyperactivation in the spinal trigeminal nucleus in a cat model of trigeminal nerve deafferentation (51). In rats, IC stimulation induced analgesia that was partially associated to aversive responses (52). Moreover, IC stimulation inhibited nociceptive neurons in the rat medullary dorsal horn, suggesting that its inhibitory nociceptive effect may occur *via* second-order neurons, following the activation of corticofugal fibers (53).

### Insula

The insula is a key integration center of nociceptive stimuli, representing an interface between sensory and limbic systems (54). Neuronal tracing studies in primates has identified the posterior insula as an important relay in the spinothalamic pathway *via* connections with the VPM (54–57). Furthermore, the posterior insula is considered an important cortical center for the integration of pain and interoception (54, 58–63).

In addition to its role as a relay in ascending pain pathways, the posterior insula is densely connected with the anterior insula, which projects to (i) brainstem nuclei involved in the descending pain modulation system (raphe, locus coeruleus, ventral tegmental area, and PAG), and (ii) limbic structures involved in the emotional valence to painful stimuli, such as the ACC and the amygdala (61, 62, 64). Intracranial recordings during painful stimuli have demonstrated the existence of nociceptive neurons in the posterior insula (54, 65). In addition, most chronic pain syndromes are associated with abnormal functional connectivity of the posterior insula. Finally, electrical stimulation of the posterior insula in neuropathic-pain rats induced antinociception by functionally modulating the opioid, cannabinoid and GABAergic systems in the PAG (66, 67). Along these lines, electrical stimulation of the insula has been proposed as a potential therapeutic alternative to restore maladaptive connectivity and activity in pathways mediating sensory and affective hypersensitivity in patients with chronic pain (54).

### Limbic-related structures

The limbic system includes a wide range of interconnected brain regions that process and regulate cognitive function, sensory perception, memory, emotions, and affective motivation (68). Various limbic regions participate in pain processing circuits, including the hypothalamus (68–70), amygdala, and ACC (68, 71–73).

The hypothalamus, a central structure in the hypothalamic-pituitary-adrenal (HPA) axis, is involved in mechanisms of psychiatric disorders, including depression, anxiety, and anorexia. The ventromedial hypothalamus (VMH), in particular, contributes to the processing of the affective dimension of pain (74), whereas the paraventricular nucleus (PVN) is part of the pain inhibitory system (75–78). Pain induced by electrical stimulation or chemical methods increases immediate early gene expression in the PVN (79–81).

In rodents, electrical stimulation of the PVN induced analgesia (78), decreased thermal pain sensitivity, knee inflammation and synovial neutrophilic infiltration (82). PVN electric stimulation enhanced c-Fos expression in the dorsal horn of the spinal cord, nucleus raphe magnus, locus coeruleus, and the PAG (75). Some of the analgesic effects of PVN stimulation seem to be mediated *via* the release of oxytocin from descending fibers to the dorsal horn (75–77).

As described above, the ACC is involved in affective-emotional aspects of pain processing (83–89). Long-term potentiation of excitatory synaptic transmission in the ACC sustains chronic neuropathic pain conditions and pain-related anxiety (90). In rodents, ACC stimulation inhibits activity of dorsal horn neurons induced by noxious mechanical stimuli (91, 92), reduces mechanical allodynia in neuropathic pain models (93), and the aversive response to noxious tactile stimulation (94). Some of these effects were associated with the activation of ACC inhibitory neurons, as optogenetic stimulation of these cells inhibited thalamic activity and reduced nociceptive behavior in experimental models of acute and chronic pain (95, 96). Similar to other brain structures, the ACC opioid system is critical to selectively modulate the aversive quality of noxious mechanical stimulation in neuropathic pain models (97). In rodents, ACC high-frequency stimulation reduced aversive pain responses induced by mechanical nociceptive stimuli, while low-frequency stimulation increased pain aversive behaviors (98). Optogenetic or chemogenetic stimulation of the ACC reduced abdominal hyperalgesia and pain-related anxiety in a rat model of chronic pancreatitis (99).

## Clinical aspects

Structures commonly targeted in patients with chronic pain are the PAG/PVG and sensory thalamic nuclei (11, 12) (Figure 1). Targets investigated to reduce the emotional components of pain include the ACC (13–16) and the VS/ALIC (17). DBS targets proposed in the past that are not routinely used to date include the IC (100, 101), septal region (102, 103), and medial thalamus (104–106).

The choice of surgical target in humans depends on whether patients present predominant neuropathic or nociceptive pain and the clinical condition associated with its development. As in animal models, it seems that stimulation of the sensory thalamus is more effective for neuropathic pain, whereas PAG/PVG has also been offered in the past to patients with nociceptive pain (39, 107–110).

The atlas most frequently used by functional neurosurgeons is the one by Schaltenbrand and Wahren (111, 112). Because the nomenclature of thalamic subdivisions in that atlas is the one proposed by Hassler (113), his system has been commonly used. According to Hassler, sensory modalities from lemniscal fibers innervate the ventralis caudalis nucleus (Vc) (113), which corresponds to the ventral posterior nuclei descried in animal studies (114). In most centers, target selection in the sensory thalamus is based on coordinates from the anterior-posterior (AC-PC) commissural plane and midcommissural point (12). As in preclinical models, Vc stimulation has to be delivered somatotopically, with face, arm, and leg regions represented from medial to lateral (114). PAG/PVG is largely targeted based on direct visualization, as this structure lies near the boundaries of the III ventricle and cerebral aqueduct. In addition to neuroimaging strategies, several centers use electrophysiology to establish the ideal target. In the sensory thalamus (115–118), stimulation induces paresthesias in projected fields, which may be used to define the somatotopic coverage of the region of interest. In addition, cells in the sensory thalamus tend to fire in specific patterns and respond to tactile and sensory stimuli (114). In contrast, PAG/PVG stimulation occasionally induces a sensation of warmth that may be characterized as pleasurable (100, 119, 120). Stimulation delivered to the PAG may sometimes induce a sense of anxiety and fear (100, 119, 120).

As described above, thalamic stimulation has been commonly used to treat neuropathic pain associated with stroke, spinal cord injury, multiple sclerosis, phantom limb pain, among others, whereas PAG/PVG was also used in the past to treat nociceptive pain (110). With the development of effective pharmacological and non-invasive treatment modalities for nociceptive pain, the use of DBS for these indications has rapidly declined.

The long-term outcome of DBS for the treatment of chronic neuropathic pain is quite variable, with most studies showing a response in 20%–70% of the patients (39, 100, 107–109, 118–136). In fact, the variability observed across studies in a recent systematic review did not allow the authors to conduct a meta-analysis (137). The inconsistency in reported outcomes has been attributed to multiple factors, including the treatment of different clinical conditions, technical aspects, and the selection of different brain targets. As described above, the overall impression in the field is that patients with neuropathic pain tend to do worse with the procedure. Since these patients predominate over those with nociceptive pain in recent studies, outcome in some recent trials tends to be worse than that of older studies. Another aspect is that patients may lose clinical benefit over time. Reasons to explain this phenomenon are unclear but may involve neuroplasticity and tolerance (100, 110, 138). The largest open-labeled multicenter trials in the field were conducted by one of the manufacturers of DBS systems (Medtronic) (139). Both studies were negative and showed a relatively poor outcome with a higher than 50% improvement being reported in 16%–47% and 13%–18% at 12 and 24 months, respectively (139).

Additional DBS targets investigated in recent clinical trials are the ACC and VS/ALIC. Following ACC stimulation, pain scores were improved by 60% and 43% at 6 and 12 months, respectively (13). Of note, ACC DBS not only induced an analgesic effect, but also improved affective components of pain (13, 14). As for the VS/ALIC, a recent randomized clinical trial in patients with poststroke pain found no difference between active or sham stimulation on the Pain Disability Index (primary outcome variable), but revealed a significant improvement in outcome measures related to the affective sphere of pain (17).

As DBS is a surgical procedure, it is important to describe potential adverse effects. There is a 2%–3% risk of intracranial hemorrhages, mostly asymptomatic. The surgery involves the implantation of hardware (electrodes, extension cables and pulse generators) (6, 39, 108, 109, 130, 140) and lead problems occur in 4%–5% of the patients. Infections may occur in 3%–5% of the patients. In approximately 50% of these subjects, parts or the entire system may need to be removed (141). Although not particularly considered a side effect, some patients implanted with Vc electrodes do not tolerate stimulation-induced paresthesias. As described above, PAG DBS has been associated with the development of stimulation-induced anxiety (110, 120, 130). A recently reported complication of ACC DBS involves the development of afterdischarges and seizures, which can be controlled by changing stimulation settings (13, 142).

## Conclusions and future perspectives

As described above, optimal DBS targets for the treatment of chronic pain are still under investigation. Possible explanations for the variable results of different preclinical and clinical studies include the complex nature of pain, the study of different conditions leading to the development of pain, and the potential involvement of multiple networks. In recent years, the concept of a traditional pain matrix has been complemented by that of a pain connectome (143). This is because pain has various salient qualities, being influenced by attention, mood and cognitive aspects (143). For example, attention-demanding tasks and stimuli can alter the quality and salience of pain and the processing of nociceptive input (144–146). In addition, connectomes can take into account the intrinsically dynamic and fluctuating relations of multiple brain structures that play a role in different aspects of pain and cognitive processes (147). Recent studies suggest that human networks constantly change over time, presenting a dynamic repertoire of brain states, including those relevant to pain and attention (148–150). Three brain systems are critical components of the pain connectome: The first is the salience network, comprised of the anterior insula, mid-cingulate cortex, temporoparietal junction, and dorsolateral prefrontal cortex (151, 152). The second system is the default mode network (DMN), which is active when subjects are instructed not to think about something specific (153). The DMN is comprised of the posterior cingulate cortex, precuneus, medial prefrontal cortex (PFC), lateral parietal lobe, and areas within the medial temporal lobe. The third system is the descending pain modulatory system and includes the PAG (143, 145, 154, 155).

The thalamus and PAG are part of the canonical analgesic pathway. The somatotopically organized sensory thalamus receives input from wide-dynamic range and nociceptive-specific neurons from the dorsal horn of the spinal cord and projects to somatosensory cortical regions (156). The disinhibition of the PAG leads to a release of norepinephrine and serotonin from the locus coeruleus and raphe into the dorsal horn, dampening pain transmission (157). Considering that high frequency DBS inhibits neuronal activity (158–160), one of the potential analgesic mechanisms of this therapy could be related to a decrease in glutamatergic transmission from thalamic cells projecting to the primary sensory cortex or a reduction in GABAergic PAG firing, followed by the disinhibition of analgesic descending pathways. This would be in line with a stimulation-induced modulation of somatosensory pain processes. In addition, chronic pain is associated with important affective-emotional components that result from the activation of the dynamic pain matrix, which involves several brain areas. The anterior limb of the internal capsule is considered a pivotal structure that interconnects limbic regions, thalamic nuclei, the ACC and PFC (161). In addition to the inhibition of neuronal cell bodies, DBS has been shown to activate neuronal appendages (i.e., axonal projections and dendrites) (7, 158, 160). Because the ALIC is predominantly composed by prefrontal/orbitofrontal-subcortical projections (161–163), stimulation of some of its limbic connections may regulate affective and emotional components of pain. Of note, ALIC DBS has been approved for the treatment of obsessive compulsive disorders and is under investigation for major depressive disorder (164–166). Another important structure in the pain neurocircuitry and connectome is the ACC, as it plays a role in depressive- and anxiety-like behaviors (167). The ACC is interconnected with the PFC, thalamus and amygdala, a set of structures rich in opiod receptors that are associated with emotional-affective deficits in individuals with chronic pain (168–170). Therefore, it is conceivable that the analgesic effects of ACC-DBS may be, at least in part, attributed to the modulation of ACC-amygdala opioid pathway. Amygdaloid projection to the PVN (171) have been shown to mediate pain transmission *via* the release of oxytocin into the dorsal horn (76, 77, 172) or following the activation of the locus coeruleus and raphe (173, 174). These data suggest that the pain matrix and connectome may be modulated through the stimulation of different structures, which may not only be involved in somatosensory, but also in cognitive and emotional aspects of pain.

In the clinic, not many DBS targets have been systematically investigated in patients with chronic pain. It is possible that some of the additional structures proposed in preclinical studies may yield more pronounced analgesic effects. A question that remains unanswered is whether any specific target is better than the other or if potential synergistic effects may occur. It is possible that a combination of targets affecting different aspects or hubs in the pain connectome may induce summative effects and more robust responses (e.g., habenula, PFC, thalamus, PAG/PVG, nucleus accumbens). This question can be potentially addressed in preclinical models using a battery of tests to investigate multiple domains. Also to be examined in preclinical models is the contribution of additional DBS mechanisms for the analgesic effects of this therapy, which go beyond the activation and inhibition of neuronal cell bodies and fibers (175). These include multiple forms of neuroplasticity, metabolic changes, neurotransmitter release, structural receptor changes, among other (7, 175, 176).

The outcome of DBS for the treatment of chronic pain is quite variable. At present, reasons for this variability have been speculated, but not clearly established. In some recently published series and in two multicenter trials, outcome was worse than the one reported in older studies (119, 135, 177). Despite the relatively small number of patients who benefit from the procedure, it seems that responders derive substantial benefit from DBS (119). With that in mind, an aspect that needs to be addressed is the development of potential biomarkers of treatment response.

As some of the effects of PAG/PVG DBS in preclinical models are mediated by endorphins, the clinical use of the morphine-naloxone test has been advocated (100, 120, 126, 178). During this test, patients are given morphine, followed by naloxone. Individuals who experience a substantial recurrence of symptoms are considered to have a prominent nociceptive component and amenable to PAG/PVG DBS. That said, the validity of this test is still unclear (179). As described above, older studies have shown a good postoperative DBS response, particularly when stimulation was delivered to the PAG/PVG, in patients with nociceptive pain. In general, nociceptive pain has a better response to opioids than neuropathic pain (180). In rodents, PAG/PVG stimulation modulates endogenous opioid transmission, suggesting a potential opioid-mediated mechanism for the antinociceptive effect of stimulation in this target. Though pain involves brain processes, the effects of DBS delivered to different regions on multiple neural circuits and neurotransmitter systems may help to explain the variable response of this therapy in patients with neuropathic and nociceptive pain.

One factor that seems to be related to a good postoperative outcome is the clinical condition leading to the development of pain. Patients with brachial plexus avulsion, complex regional pain syndrome, and peripheral neuropathy seem to have a better response to DBS than those with postherpetic neuralgia or thalamic pain (39, 108, 109, 130, 135). Psychological or litigation problems forecast a poor prognosis (39, 110, 130). Preclinical work could help to address some of these aspects, as animals, in theory, do not have a strong psychosocial overlay. That said, the effects of cognitive, stress and depression-like behaviors in animal models of nociception have been poorly explored. This field of research could certainly be expanded, since DBS delivered to different targets has been shown to induce antidepressant-, antianhedonic-, and anxiolytic-like effects in rodents (7, 159, 181). To more closely mimic the multiple components of pain in humans and increase its translational value of preclinical studies, models could include not only nociceptive assessments, but a battery of paradigms to evaluate cognitive and psychiatric-like behaviors as well.

Another factor suggested to forecast a positive response to invasive neuromodulation procedures is the so-called insertional effect, characterized by the amelioration of pain immediately after electrode implantation in the absence of stimulation (119, 182). This may also explain the better results observed in preclinical models compared to humans, as the ratio between the electrode diameter and target volume is far more pronounced in rodents.

In a recent series of studies in patients implanted with both Vc and PVG electrodes, field potentials were recorded in the thalamus during stimulation of the latter (183, 184). A decrease in low frequency thalamic potentials after PVG stimulation was found to predict a good therapeutic response to DBS (183, 184).

Despite the complex nature of pain and the fact that animal models do not reflect the subjective nature of this condition, multiple preparations have emerged over the years. These are rooted in dimensions of face, construct and predictive validity (7, 18, 159). The former refers to similarities between the model and clinical symptoms. Construct validity reflects neurobiological similarities between the model and the human condition. Predictive validity reflects commonalities in treatment response between patients and the preclinical scenario. Our study confirms the predictive validity of animal models, as DBS delivered to clinically relevant targets in animals reduces nociception. Compared to the vast clinical literature, a limited number of studies have been published in animal models. With the translational potential described above and the well-described mechanisms of nociception, work in preclinical models is certainly underutilized in the field of DBS for pain. Additional studies using modern neuroscience techniques could unravel the mechanisms and neurocircuitry involved in the analgesic effects of DBS and help to optimize this therapy. These could include the use of batteries of tests to measure the effects of DBS in different behavioral domains, the use of connectivity analyses, the stimulation of multiple brain targets at the same time, or the co-treatment of animals with DBS and different medications to assess whether certain classes of drugs may potentiate the effects of stimulation (7, 185, 186). In addition, chemogenetics, optogenetics and other molecular techniques can be used to deconstruct and dissect the neural circuits and cells involved in the mechanisms of DBS.
